# Higher-order theories of consciousness and what-it-is-like-ness

**DOI:** 10.1007/s11098-017-0980-8

**Published:** 2017-10-06

**Authors:** Jonathan Farrell

**Affiliations:** University of Manchester, 49 Kings Lane, Stretford, Manchester M32 8GG UK

**Keywords:** Higher-order theories of consciousness, What-it-is-like-ness, Consciousness, ‘What it is like’, ‘Something it is like’, Awareness, Subjectivity, Misrepresentation, Rosenthal, Phenomenal consciousness, Conscious experience

## Abstract

*Ambitious* higher-order theories of consciousness aim to account for conscious states when these are understood in terms of what-it-is-like-ness. This paper considers two arguments concerning this aim, and concludes that ambitious theories fail. The *misrepresentation argument* against HO theories aims to show that the possibility of radical misrepresentation—there being a HO state about a state the subject is not in—leads to a contradiction. In contrast, the *awareness argument* aims to bolster HO theories by showing that subjects are aware of all their conscious states. Both arguments hinge on how we understand two related notions which are ubiquitous in discussions of consciousness: those of *what*-*it*-*is*-*like*-*ness* and *there being something it is like for a subject to be in a mental state*. This paper examines how HO theorists must understand the two crucial notions if they are to reject the misrepresentation argument but assert the awareness argument. It shows that HO theorists can and do adopt an understanding—the *HO reading*—which seems to give them what they want. But adopting the HO reading changes the two arguments. On this reading, the awareness argument tells us nothing about those states there is something it is like to be in, and so offers no support to ambitious HO theories. And to respond to the misrepresentation understood according to the HO reading is to simply ignore the argument presented, and so to give no response at all. As things stand, we should deny that HO theories can account for what-it-is-like-ness.

## HO theories of consciousness

“Consciousness” as Thomas Nagel noted, “is what makes the mind–body problem really intractable.” (1974, p. 435) One way to gain traction is to divide the problem in two: first explain consciousness in terms of representation; then give a physicalist account of representation. We can make the first step, some claim, by adopting a higher-order theory of consciousness. This paper argues that such theories cannot make this first step (it is not concerned with the second step at all).

Higher-order (henceforth ‘HO’) theories of consciousness say, roughly, that a state, M, is conscious just when its subject, S, has a HO mental state which is about M. The HO state must not arise (or must not seem to arise) via inference or observation, and the HO state is about M in the sense that it represents M as being some way. Although specific HO theories differ in what sort of mental state they take the HO state to be—a perceptual (or perceptual-like) state,^1^ a thought,^2^ or a non-occurrent state^3^—these differences will not concern us here.

To judge whether a theory succeeds, we need some pre-theoretical grasp of what the theory aims to explain. HO theories of consciousness aim to account for conscious *states*. And one way in which a mental state can be said to be conscious is when the subject is conscious (i.e., aware) *of* that state. This understanding is captured by the *Transitivity Principle* (where M is a mental state and S a subject):

TP   M is conscious *only if* S is aware *of* M^4^

Another way we can understand a state’s being conscious is in terms of the notion of “what-it-is-like-ness”. It is consciousness in this sense which Nagel is concerned with in the paper quoted above. This notion of consciousness is captured by the popular *Nagelian Definition*:

ND    M is conscious *iff* there is something it is like for S to be in M^5^ ^6^

There are other ways of making sense of a state’s being conscious. In particular, some philosophers appeal to the notion of a state’s being *phenomenally conscious*. Often, this is closely tied to the idea of a state’s being conscious in the what-it-is-like sense, but sometimes this connection is resisted.^7^ I will remain agnostic on the relation between what-it-is-like-ness and phenomenal consciousness. This paper is concerned with consciousness as understood in terms of TP, consciousness as understood in terms of ND, and how they are related.

HO theories are well-placed to account for states that are conscious in the TP sense. One way in which we can be aware of a mental state, M, (as required by TP) is by having a HO thought which represents M as being some way. Importantly, if we understand TP in this way (as I henceforth will) it doesn’t require that S be in M, or that M even exist: that M is conscious in this sense is a matter of how M (mentally) *appears* to be to the subject, not how M *is*. Whether HO theories can account for what-it-is-like-ness, however, is controversial. Some “modest” HO theorists don’t aim to provide such an account. “Ambitious” theorists, on the other hand, do, and it is with ambitious theories that we will be concerned here.^8^

I’ll consider two arguments relevant to ambitious HO theories. The *misrepresentation argument* aims to show that these theories fail because they allow for cases of radical misrepresentation: a HO state can represent the subject as being in a state they are not in. In contrast, the *awareness argument* supports HO theories. This argument aims to show that what-it-is-like-ness requires that subjects be aware of their conscious states, a fact which HO theories are particularly well placed to account for. Both arguments hinge on how we understand the related notions of:

Something    There is something it is like for S to be in M

What    There is an occurrence of what-it-is-like-ness associated with M.^9^

Thus to assess the arguments—and so to assess the viability of HO theories of consciousness—we will need to look at these notions in more detail.

In Sects. 2 and 3 respectively, I will describe the misrepresentation and awareness arguments and show how, if HO theorists are to reject the former but assert the latter, they must understand what and something.^10^ I’ll also show that this requires HO theorists to understand these notions in non-standard ways. In Sect. 4 I’ll show that HO theorists do understand these notions in this way and so it seems as if they can respond to the misrepresentation argument while asserting the awareness argument. But, as I’ll argue, things are not as they prima facie seem to be. If we adopt the HO reading of what and something then the awareness argument is uninteresting and offers no support to ambitious HO theories. And although HO theorists can respond to the misrepresentation argument when this is understood according to the HO reading, this doesn’t help them respond to the misrepresentation argument as it is intended to be understood, which is not according to the HO reading.^11^

It is perhaps worth stressing that, although this paper focuses on what we might call semantic or conceptual matters, the ultimate topic of interest is consciousness, not ‘consciousness’. The knowledge and conceivability arguments against physicalism each rely on claims about epistemology but aim to deliver a metaphysical conclusion. Discussing the relevant epistemic matters is not to turn away from questions about the nature of consciousness. Likewise, the two arguments considered here hinge on claims about how we understand something and what, but that doesn’t mean that their conclusions—or the conclusions of this paper—are not about consciousness but merely concern words or concepts.

## The misrepresentation argument

One objection to HO theories stems from the fact that these theories allow for the possibility of misrepresentation.^12^ A misrepresentation case occurs when the HO state represents M as being some way even though the subject is not even in M.^13^ The *misrepresentation argument* assumes a misrepresentation case and deduces a contradiction, as follows:

(M1)    M is conscious & S is not in M

(M2)    If M is conscious, there is something it is like for S to be in M

(M3)    If there is something it is like for S to be in M, there is an occurrence of what-it-is-like-ness associated with M

(M4)    If there is an occurrence of what-it-is-like-ness associated with M, S is in M

(M5)    If there is something it is like for S to be in M, S is in M. (From (M3, M4).)

(M6)    S is in M. (From (M1), (M2), (M5).)

(M7)    S is not in M. (From (M1).)

(M8)    S is in M & S is not in M. (From (M6), (M7).)^14^

Since we end up with a contradiction, we must reject one of the premises of the argument. But, the reasoning goes, (M2) follows from ND, the Nagelian definition of consciousness, and so can’t be rejected on pain of changing the subject and talking about something other than what-it-is-like consciousness (and so abandoning an ambitious HO theory). (M3) follows from the very notion of there being something it is like for S to be in M, i.e., from something. (M4) follows from the very notion of there being an occurrence of what-it-is-like-ness, i.e., from what (rejecting it “amounts to abusing the notion of what-it-is-like-ness” (Block 2011, p. 427)). So we must reject (M1) and deny that misrepresentation cases are possible. Thus HO theories—which allow that such cases are possible—cannot account for what-it-is-like-ness.

HO theorists aim to reject the misrepresentation argument by rejecting (M5). Since (M5) is entailed by (M3) and (M4), this requires denying one of these premises. One response is for HO theorists to emphasise that they understand something and what in terms of a subject’s being aware of a mental state.^15^ Presumably the counter-argument goes something like this:

(N1)    If S is aware of M, then there is an occurrence of what-it-is-like-ness associated with M

(N2)    It is not the case that: if S is aware of M, then S is in M

(N3)    It is not the case that: if there is an occurrence of what-it-is-like-ness associated with M, S is in M. (From (N1), (N2).)

This argument is valid, and (N3) is the negation of (M4). But (a) is suspect: it’s not generally true that a subject’s being aware of a mental state entails that there is an occurrence of what-it-is-like-ness associated with it (and the converse of (a) delivers an invalid argument).^16^ Perhaps HO theorists can finesse the notion of awareness involved, but we do not need to consider this now. This is because our current aim is to establish how HO theorists must understand something and what if they are to reject the misrepresentation argument. That HO theorists understand TP-consciousness as a matter of mental appearances as noted above (Sect. 1), and exactly how they understand this awareness, or that involved in what-it-is-like consciousness, does not matter here since the notion of awareness plays no role in the misrepresentation argument.^17^ We return to awareness in Sect. 3.^18^

How, then, can HO theorists reject either (M3) or (M4)? These premises are intended to follow from the very notions of something and what. To see how the premises can be rejected requires examining how the notions might be understood. I’ll consider each premise in turn.

### (M3): Non-occurrent and occurrent readings of something

We might think that (M3) is the premise to reject since it looks to be false: it is not true that, just because there is something it is like for S to be in M, there is an occurrence of what-it-is-like-ness associated with M. After all, before Mary the super-scientist in Jackson’s famous knowledge argument (1982) leaves her a black and white room and sees her first coloured object she is (according to the story) ignorant of something: she does not know what it is like for her to, say, see red. This is not because there is nothing it is like for her to see red: as she sits in her room, there is something it is like for her to see red. Mary is ignorant because she doesn’t know what it is like for her to see red. Thus ‘there is something it is like for Mary to see red’ can be true in situations when there is no occurrence of what-it-is-like-ness associated with seeing red.^19^

To understand the knowledge argument as it is intended, then, requires adopting what I’ll call a *non*-*occurrent* reading of something.^20^ On such a reading, there being something it is like for S to be in M at some time does not entail that, at that time, there is an occurrence of what-it-is-like-ness associated with M. All it requires is that, when S is in M (e.g., when Mary sees red), there is such an occurrence (e.g., one of what-it-is-like-ness associated with seeing red). We can contrast this reading with an *occurrent* reading of something. On this reading, there being something it is like for S to be in M *does* entail that there is an occurrence of what-it-is-like-ness associated with M, but it does not entail that there is such an occurrence any time S is in M.^21^ The reading of something as it features in the knowledge argument seems to be the standard one. None of the familiar responses to this widely discussed argument are of the form: ‘Jackson’s use of “what it is like” and “something it is like” is idiosyncratic.’ So consideration of the knowledge argument gives us one reason to think that the standard reading of something is the non-occurrent one.^22^

A second reason for thinking this is that it is natural to explain why we seek out some situations and avoid others by employing the notions something and what. One of the reasons why I try to avoid having a migraine is that there is something it is like for me to have a migraine, and the occurrence of what-it-is-like-ness associated with a migraine state is unpleasant. But on the occurrent reading of something, since I am not now having a migraine, it is not true that right now there is something it is like for me to have a migraine. Thus the “explanation” just given is no explanation at all. But surely this is a mistake: the explanation *is* a good one, and that it is shows that we standardly adopt the non-occurrent reading of something.

A third reason for thinking that the non-occurrent reading is standard is that, when we look at other English sentences which have a similar form to something, we can see that we adopt the non-occurrent reading of them. The truth now of ‘There is some time it takes for Rihanna to run a mile’, for example, doesn’t require that Rihanna is now running (or has ever, or will ever, run) a mile. What matters is that, when she runs a mile (or were she to run one), it takes (or would take) her some time to do so. These three considerations show that the non-occurrent reading of something is the standard one. But to accept (M3) requires adopting the non-standard, occurrent reading. Thus, we might think, HO theorists can easily reject the misrepresentation argument by understanding something in the standard way.

But HO theorists cannot do this because their theories commit them to the non-standard, occurrent reading of something. HO theories are *extrinsic* theories of consciousness (Weisberg 2011b). Such theories hold that consciousness is an extrinsic property of states—what determines whether a state is conscious involves something distinct from the state itself (e.g., the presence of an appropriate HO state). On non-occurrent readings of something, to say that there is something it is like for S to be in M is to say that *when* S is in M, S undergoes phenomenology associated with M. This cannot be true if what determines whether S undergoes phenomenology associated with M depends, in part, on something other than M: S can be in M without the extra factor being present.

Further, as HO theorists understand something, there being something it is like for S to be in M requires that there be an appropriate HO state about M, and this in turn suffices for there being an occurrence of what-it-is-like-ness associated with M. But as our discussion of Mary shows, on the standard reading of something, there being something it is like for Mary to see red doesn’t entail that there is an occurrence of what-it-is-like-ness associated with seeing red. Thus HO theorists are committed to (M3) and must reject a different premise if they are to successfully respond to the misrepresentation argument.^23^

### (M4): Tight and loose readings of what

Proponents of the misrepresentation argument understand what in a way which guarantees the truth of (M4) (to do otherwise, recall, “amounts to abusing the notion of what-it-is-like-ness”):

(M4)    If there is an occurrence of what-it-is-like-ness associated with M, S is in M

I’ll call a reading of what which delivers (M4) a *tight* reading since it commits us to there being a tight association between, on the one hand, there being an occurrence of what-it-is-like-ness associated with M, and, on the other, S being in M. We can see the attractiveness of the tight reading if we consider being in pain. If there is an occurrence of what-it-is-like-ness associated with pain, we might say, one *just is* in pain: that’s what it is to be in pain.^24^ On the tight understanding of what, then, you can’t undergo what-it-is-like-ness associated with M, unless you are in M.^25^

If HO theorists are to reject (M4), then they must adopt a *loose* reading of what. On a loose reading, S doesn’t need to be in M for there to be an occurrence of what-it-is-like-ness associated with M. One way this could be so (the way favoured by HO theorists) is if we understand there being an occurrence of what-it-is-like-ness associated with, say, having a migraine as an occurrence of representing oneself as having a migraine. Clearly, this representing can occur without the migraine occurring. So if HO theorists adopt a loose reading of what (as Rosenthal (2011) and Weisberg (2011a) plausibly do when they reject Block’s accusation of abuse), they can reject (M4), and with it the misrepresentation argument.

Whether we adopt a tight or loose reading of what is in principle orthogonal to whether we adopt an occurrent or non-occurrent reading of something. But the standard, non-occurrent reading of something fits naturally with the tight reading of what. On the non-occurrent reading of something, when S is in M, there is an occurrence of what-it-is-like-ness. A natural explanation of why this is so is that there is a close connection between the what-it-is-like-ness associated with M and S’s being in M. But this is just to adopt the tight reading of what. Since the non-occurrent reading of something is the standard reading, and this fits naturally with the tight reading of what, this suggests that the tight reading of what is the standard one.

To resist the misrepresentation argument, HO theorists must adopt an occurrent reading of something and a loose reading of what. In Sect. 4 we’ll see that this is indeed what HO theorists do. First, however, we must note another constraint on how HO theorists must understand these two notions.

## The awareness argument

The *awareness argument* aims to show that HO theories are better placed to explain what-it-is-like consciousness than some rival views. The argument is simple.

(A1)    If M is conscious, then there is something it is like for S to be in M

(A2)    If there is something it is like for S to be in M, then S is aware of M

(A3)    If M is conscious, then S is aware of M. (From (A1), (A2).)^26^

The conclusion of this argument links consciousness in the what-it-is-like sense with consciousness as captured by the transitivity principle. Something like (A3) is endorsed by many philosophers.^27^ It does not, however, garner universal assent.^28^ Thus although the claim seems obvious to some, those who accept it need to provide some reasons in favour of it, and that is just what the awareness argument does. If this argument succeeds, theories of consciousness must accommodate the fact that we are aware of our conscious states. If conscious states are indeed, as HO theories say, those we represent ourselves as being in, then this either involves, or requires only a short step to reach, the idea of the subject being aware of the state. So HO theories are clearly well placed to accommodate (A3). Some rival theories of consciousness such as first-order theories of consciousness—which say, roughly, that conscious states are those states we are conscious *with* 29—must do more work if they are to account for the feature of conscious states (A3) highlights.

The awareness argument is valid, and (A1) follows from the widely accepted Nagelian definition, ND. Thus we should accept the argument only if we accept premise (A2). Why do HO theorists think we should accept (A2)? It cannot be because they take (A2) to be a version of the transitivity principle, TP, i.e., that there being something it is like for S to be in M *just is* M’s being TP-conscious.^30^ This is because the awareness argument is offered as support for something like this identity claim. Assuming it in order to accept (A2) begs the question.

Similarly, HO theorists can’t accept (A2) simply because they think it best explains what distinguishes states that are conscious in the what-it-is-like sense from those that are not. Why think this is the best explanation? The obvious answer is: because we distinguish states that are TP-conscious from those that are not by noting that we are aware of the former, and there is a close connection between TP consciousness and what-it-is-like consciousness. But, again, assuming that this close connection holds means assuming what is trying to be shown: that ambitious HO theories of consciousness succeed.

To support an ambitious HO theory by way of the awareness argument requires explaining why we should accept (A2) without assuming a tight connection between what-it-is-like and TP consciousness. Proponents of the awareness argument give us such a reason: we should accept (A2) because it follows from the meaning of its antecedent, i.e., of something. Rosenthal says that “in any *sense of the phrase* ‘what it’s like’ that has any bearing on consciousness[, w]hen one lacks conscious access to a state, there is literally nothing it’s like for one to be in that state.” (2000, p. 275, my emphasis). Weisberg claims that “*the ‘for’ stressed by Nagel is crucial: the notion indicates* a subjective awareness of an organism’s mental states by the organism itself.” (2011a, p. 439, my emphasis). And Janzen says that, “*the very language of the what*-*it*-*is*-*like formula, the words in it*, suggests that it ought to be read as expressing a proposition about a subject’s awareness of her own mental states.” (Janzen 2011, p. 283, my emphasis). Kriegel also stresses the ‘for’ in something and suggests that it doesn’t *make sense* to deny (A2): it is “quite possibly incoherent.” (2009, p. 105). The awareness argument, then, hinges on how we understand something.

### (A2): Self-intimating and non-self-intimating readings of something

On what I’ll call a *self*-*intimating* reading of something, there being something it is like for S to be in M entails that S is aware of M. We can resist the awareness argument by instead adopting a *non*-*self*-*intimating* reading of something. On this reading, something does not entail that S is aware of M (it does not follow from something, on this reading, that S is *not* aware of M).

Which of these readings of something we should accept is independent of whether we adopt an occurrent or non-occurrent reading of something, and of whether we adopt a tight or loose reading of what. But the self-intimating reading of something does not fit well with the standard, non-occurrent reading of something. On the latter, there being something it is like for S to be in M does not entail that there is an occurrence of what-it-is-like-ness associated with M. This sits ill with it being the case that S is aware of M, as is required by the self-intimating reading of something. To go back to Jackson’s Mary, before she leaves her room there is (on the standard reading) something it is like for Mary to see red, but Mary is not then aware of the mental state of seeing red.^31^ The self-intimating reading of something is far more plausible if we adopt the non-standard, occurrent reading, which suggests that the self-intimating reading itself is non-standard.

For the awareness argument to succeed, then, requires that we adopt the self-intimating—i.e., the non-standard—reading of something. To do so doesn’t beg any questions: the awareness argument aims to show a close connection between TP-consciousness and what-it-is-like consciousness, but there being such a connection doesn’t entail any claims about how we *understand* phrases such as ‘something it is like’. Should we adopt the self-intimating reading? Proponents of the awareness argument argue that we should, but, as I’ll now show, their arguments are not convincing.

### Arguments for the self-intimating reading

Weisberg effectively defends the self-intimating reading of something when he claims that “the ‘for’ stressed by Nagel is crucial: the notion indicates a subjective awareness of an organism’s mental states by the organism itself.” (Weisberg 2011a, p. 439)^32^ But he offers no reason why should we think that this is what this ‘for’ means. Appealing to dictionaries doesn’t help here: they don’t include such a meaning.^33^ Since we’re given no reason to think that we should understand ‘for’ in this way, the first argument for the claim that we should adopt a self-intimating reading of something is unpersuasive.

A second argument can also be found in Weisberg (2011a). The idea here is that a self-intimating reading is “moderate” and so should be preferred to a rival “zealous” reading.^34^ On the “zealous” reading, something does not entail that S is aware of M (i.e., it is a non-self-intimating reading), but it does entail that consciousness is a monadic property of mental states. In contrast, the “moderate” reading is a self-intimating reading, but something entails nothing about the nature of consciousness. Since it involves fewer commitments, Weisberg claims, we should prefer the “moderate” reading. It’s not obvious that we should accept this “fewer commitments” principle, but even if we do, the argument fails since it presents a false dilemma. Consider a *third* reading which is neutral both about awareness (i.e., is non-self-intimating) and also about the nature of consciousness. The “fewer commitments” principle says we should prefer this—a non-self-intimating reading—to either the “zealous” or “moderate” readings.

The third argument for the claim that we should adopt a self-intimating reading of something—the *provenance argument*—has two premises.^35^ The first is that when philosophers appeal to the notion of something, they understand it in the same way as Nagel did in his ‘What is it like to be a bat?’ (1974). The second is that Nagel adopted a self-intimating reading of something. It is undeniable that the popularity of appealing to the notion of there being something it is like for a subject to be in a state in discussions of consciousness is due in large part to Nagel’s paper.^36^ And we can assume that—unless they note otherwise—those who invoke this notion are not aiming to change the subject: they intend it to be understood in roughly the same way that Nagel understood it. Thus the first premise is true.

What about the second premise— that Nagel holds a self-intimating reading of something? Janzen gives two arguments for this premise. The first appeals to Nagel’s use of “the Sartrean terms ‘pour-soi’ (the ‘for-itself’) and ‘en-soi’ (the ‘in-itself’)” (2011, p. 284) in his (1974). Since Sartre holds that we are always aware of our conscious states, Janzen claims, we should interpret Nagel as doing likewise. But Nagel does not explain how he understands Sartre’s (French, technical) terminology (which Sartre uses to describe objects, not mental states), and uses it only once and in passing. And even if this suggests that there is *some* similarity between Nagelian and Sartrean notions of consciousness, there’s no reason to think that the similarity concerns awareness of conscious states. So Janzen’s first argument fails.

Janzen’s second argument relies on the claim that Nagel explains the meaning of something in terms of the subjective/objective distinction which he elsewhere (in his (Nagel 1965)) explains in terms of “psychological internality”. But Nagel doesn’t attempt to *explain* the meaning of something in his 1974 paper. On the contrary, he appeals to something to explain what subjective character is (1974, p. 436). Nor does Nagel use the term ‘psychological internality’ at all (and ‘internality’ only once) in his 1965 paper, so it doesn’t seem to be a central notion for him. Nor is it obvious that what Nagel refers to by ‘internality’ is to be understood in terms of awareness of conscious states. So Janzen’s second argument fails. We have no reason to accept the second premise of the provenance argument: that Nagel adopted a self-intimating reading of something.^37^ Thus the provenance argument fails.

The fourth argument for the proposition that we ought to adopt a self-intimating reading of something is the *analogical argument*. The argument (Janzen 2011, p. 283) begins with the claim that ordinary objects can be like something for us—e.g., they can look, or smell, like something. It follows from the meaning of ‘There is something O looks (or smells, etc.) like for S’ (where ‘O’ stands for an ordinary object) that S is aware of O. Thus we reach a subsidiary conclusion: if O is like something to S, S is aware of O. Next the analogical claim is made: what goes for ordinary objects also goes for mental states. So, if there is something M is like for S, then S is aware of M. Since ‘there is something M is like for S’ is true just when something is, the argument establishes that if there is something it is like for S to be in M, then S is aware of M. That is, it establishes that the correct reading of something is a self-intimating one.

The analogical argument fails because ‘There is something M is like for S’ does not have the same truth conditions as ‘There is something it is like for S to be in M’—i.e., as something. Presumably we are meant to think it does because these are just two ways of saying the same thing. And we’re meant to believe this because these two sentences are mere grammatical rearrangements of each other: although they are distinct sentences, the rules of English grammar allow us to transform one into the other without change in meaning. This is so in just the way that ‘Adam loves Eve’ and ‘Eve is loved by Adam’ are grammatical rearrangements of each other, as are ‘It’s embarrassing that he is drunk’ and ‘That he is drunk is embarrassing.’

But ‘There is something M is like for S’ is not a grammatical rearrangement of something as we can see by considering similarly structured sentences.

(a)    There is some time it takes for Rihanna to run a mile.

is a rearrangement of

(b)    There is some time running a mile takes for Rihanna.

To make the rearrangement, we take the infinitive verb phrase from (a) (‘to run a mile’), change the verb to the ‘-ing’ form (‘running a mile’), and then move this phrase from the end of the sentence into the location of ‘it’, giving us (b). If we apply these rules to ‘There is something it is like for S to be in M’, we take the phrase ‘to be in M’, change ‘be’ to ‘being’, and move the verb phrase into the location occupied by ‘it’, producing

(c)    There is something being in M is like for S.

Clearly, (c) is not the sentence involved in the analogical argument, which is

(d)    There is something M is like for S.

Nor can we get (d) by rearranging (c): (c) does not mean what (d) means. If (c) did mean what (d) means then the meaning of ‘being in M’ in (c) would have to be the same as that of ‘M’ in (d), but it is not. The analogical argument, then, depends on a claim—that (d) is a grammatical rearrangement of something—which is false.

We might hope to fix the argument by amending the analogical premise so that we rely on the (true) claim that something is a grammatical rearrangement of (c). This requires that what we have on the “mental” side of the analogy is *being in a mental state*, rather than *a mental state*. But then what is on the “object” side of the analogy? There seem to be three options. First, that *being in a mental state* is analogous to *an ordinary object*. But this is implausible: these are very different kinds of things. Nor is the second option—that *being in a mental state* is analogous to *being in an object*—plausible: the second ‘in’ indicates spatial containment, the first does not. The third option is that it is *perceiving an object* which is analogous to *being in a mental state*. But what is analogous to the former in the mental realm is surely *perceiving*—not *being in*—*a mental state*. There is no plausible analogical claim that can get us where the analogical argument needs to go. Thus the argument fails.

We’ve examined four attempts to show that we ought to adopt a self-intimating reading of something, and all four fail. We have no reason to revise our view that it is the non-self-intimating reading of something which is the standard one.

## The HO reading of something and what

If ambitious HO theorists are to resist the misrepresentation and affirm the awareness arguments they must hold that if something is true then S is now undergoing what-it-is-like-ness associated with M, that S is aware of M, and that S’s undergoing what-it-is-like-ness associated with M does not require that S be in M. In other words, they must adopt an occurrent, self-intimating reading of something and a loose reading of what. How do ambitious HO theorists understand these notions?

I’ll take Rosenthal to be representative when he says, “As many, myself included, use that phrase, there being something it’s like for one to be in a state is simply its seeming subjectively that one is in that state.” (Rosenthal 2011, p. 433)^38^ And, as Rosenthal understands ‘seeming subjectively’, when it seems subjectively to us that we are in some state, M, we represent ourselves as being in M. We do this by way of being in a HO state (for Rosenthal, a thought) which is about M. What it is like for S to be in M, on this understanding, is just how S represents M as being: what it is like for me to have a migraine is just how I represent my migraine state to be.

This gives us what I’ll call the *HO reading* of something and what. On this reading of something, there is something it is like for S to be in M just when S is in a HO state which represents M. And there being an occurrence of what-it-is-like-ness associated with M is just there being some way that S represents M as being—i.e., S’s being in a HO state which represents M. Thus on the HO reading, the notions of something and what are very closely linked: there is something it is like for S to be in M just when there is an occurrence of what-it-is-like-ness associated with M, i.e., just when S has a HO state which represents M.

We can see that the HO reading is just what ambitious HO theorists need. It is occurrent: there being something it is like for S to be in M entails that there is an occurrence of what-it-is-like-ness associated with M. It is a self-intimating reading: there being something it is like for S to be in M means that S has a HO state that represents M, and so S is thereby aware of M. And it is a loose reading of what: that there is an occurrence of what-it-is-like-ness—that S represents M—is compatible with S not being in M. The HO reading is non-standard on every dimension—the standard reading is occurrent, tight and non-self-intimating—but this doesn’t mean that this reading is in some way illegitimate or unacceptable.

Adopting the HO reading means that ambitious theorists can resist the misrepresentation argument and so hold on to their ambitions. And they can assert the awareness argument which links what-it-is-like consciousness with TP consciousness. At least, this is how things initially seem, and it is how those sympathetic to HO theories take things to stand (e.g., Rosenthal (2011), Weisberg (2011a) and Shepherd (2013)). But more needs to be said.

### The arguments again

If we adopt the HO reading, the misrepresentation argument fails because premise (M4) is false. The awareness argument, on the other hand, appears sound since the crucial premise there, (A2), comes out as true. But how we understand something and what does not just affect how we understand (M4) and (A2). If each argument is to remain valid, we must adopt the HO reading throughout. In particular, we must adopt this reading when it comes to the claim which appears in both arguments (as (A1) and (M2)):

(A1/M2)    If M is conscious, then there is something it is like for S to be in M.

The reason given above (Sects. 2, 3) for accepting (A1/M2) was that it follows from the popular Nagelian definition of consciousness:

ND    M is conscious *iff* there is something it is like for S to be in M

As noted (Sect. 3.2), ND’s popularity stems from Nagel’s use of sentences like something to characterise conscious states. Thus what is popularly taken to be plausible is that, if we understand something more or less as Nagel did then ND captures something important about conscious states. If we are to adopt the HO reading throughout the misrepresentation and awareness arguments, and to motivate (A1/M2) by noting that it follows from ND, then it must be that the HO reading more or less accords with the Nagelian reading. Note that the claim here is not that Nagel has any special authority in deciding in how we understand something as it appears in ND. The claim is rather that we ought to understand something in ND in the same general way as the very many philosophers who appeal to this definition do (this is where the authority lies). And—as the popularity of appealing to such notions in discussions of consciousness in the years following Nagel’s paper shows—the way these very many philosophers understand something in ND is in roughly the way that Nagel did.

As we saw in Sect. 3.2 there is no reason to think that Nagel adopts the HO reading of something. Further, it is clear that Nagel does *not* understand something in this way. Nagel’s thesis in his (1974) is that physicalism is in trouble. One of the physicalist theories Nagel is criticising (n1, 435) is Armstrong’s (1968) HO theory of consciousness. It is just implausible, then, that Nagel thinks that there being something it is like for a subject to be in a state—the feature he thinks physicalism *cannot* account for—should be understood in terms of our being in a HO state which represents these states—a phenomena that physicalist HO theorists such as Armstrong, plausibly can (at least insofar as anything Nagel says) account for.^39^ And this should not be surprising given the discussions above: the standard way of understanding something and what are those in the Nagelian tradition. And we’ve seen that the HO reading is non-standard on every dimension.

It is true as Weisberg says, that “there is another way to interpret Nagel’s phrase” (Weisberg 2011b, p. 411) than the standard way. But to interpret the phrase as it appears in ND in this other way—a way different to that in which Nagel and the many following him who use this phrase to pick out conscious states do—is to *mis*interpret the definition, and so to change the subject. If ND as it is standardly understood is true (and the many who appeal to it surely take it to be so), then ND understood according to the HO reading is false.^40^

Our reason for accepting (A1/M2) was that it follows from ND. But if we understand (A1/M2) according to the HO reading, it doesn’t follow from ND (on the standard reading). It *does* follow from ND (on the HO reading), but on this reading ND is false, so this doesn’t help. Thus we have no reason to accept (A1/M2) understood according to the HO reading. This means that the awareness argument fails: if (A1) is to be true, we must adopt the standard, non-HO reading of something; if (A2) is to be true, we must adopt the non-standard, HO reading. But if we do this we equivocate, and so the resulting argument is invalid. A similar problem arises for the misrepresentation argument: if the argument is understood as its proponents intend then the standard reading of something must be adopted throughout. To attempt to understand the argument in accordance with a different reading—such as the HO reading—is just to change the subject, and any response to *this* argument is not a response to the misrepresentation argument opponents of HO theories present.

### A response

Ambitious HO theorists might respond to this line of thought as follows: HO theories are only committed to accounting for what-it-is-like consciousness when this is understood according to the HO reading. Whether or not HO theories can account for what-it-is-like consciousness on some other, non-HO-reading, is irrelevant.^41^ One way to put this response is to note that, in some theoretical contexts it can be useful to understand terms in a non-standard way, and to add that we are now in such a context.^42^

HO theorists are free, of course, to decide what their theory is a theory of, and to only present the awareness argument in the kind of theoretical context which favours or allows the HO reading. If this is how things stand, however, it is hard to see what distinguishes *ambitious* HO theorists—which now means those who aim to account for what-it-is-like consciousness *but only on the HO reading*—and modest HO theorists—those who only aim to account for consciousness in the sense given by the transitivity principle, TP. On the HO reading, there is no interesting difference between ND and TP given that HO theorists explain a state’s being conscious in the TP sense in terms of its being the target of an appropriate HO state. Thus there is no difference between ambitious and modest HO theories and no interesting sense in which HO theories can account for what-it-is-like consciousness.

HO theorists are not free, however, to interpret the misrepresentation argument as concerning what-it-is-like consciousness on the HO reading. As noted above, to do so is not to consider the argument they are presented with. They can, of course, say that they are only interested in what-it-is-like consciousness in those theoretical contexts in which the HO reading is appropriate. To do this, however, is just to acknowledge that they are not offering an ambitious HO theory: they do not aim to account for what-it-is-like consciousness as this is standardly understood.

## Conclusion

I have argued that ambitious HO theories of consciousness fail. The claim is not that HO theories fail because adopting a non-standard reading of what and something is somehow undesirable or unacceptable: I do not claim that there is anything problematic with the HO reading itself. Instead, they fail because responding to the misrepresentation argument as understood in accordance with the HO reading is not to respond to the argument at all: it is to offer no response to the argument that concludes that HO theorist cannot account for what-it-is-like consciousness as this is standardly understood—i.e., as relevant to that notion of consciousness which Nagel suggests makes the mind–body problem intractable. And the awareness argument—which is also advanced by self-representational theorists (see (Kriegel 2012))—only succeeds if we adopt the HO reading. But doing so fails to establish a link between what-it-is-like consciousness (as standardly understood) and consciousness as understood in terms of the transitivity principle, TP. Perhaps HO theories can account for what-it-is-like-ness in a non-standard sense, but this amounts to nothing more than accounting for TP consciousness—there is nothing ambitious about such a theory.

I also described three pairs of contrasting ways of understanding two notions central to discussions of consciousness in the literature, namely:

Something   There is something it is like for S to be in M

What    There is an occurrence of what-it-is-like-ness associated with M

Getting clear about how we understand these notions allows us to see that the misrepresentation argument against ambitious HO theories succeeds while the awareness argument in favour of them fails. Although I have not considered the question here, attending to these distinctions may help shed light on other debates concerning consciousness.

## Footnotes

1 Armstrong (1968) and Lycan (1996).

2 Rosenthal (1986) and Weisberg (2011b).

3 Carruthers (2000).

4 Rosenthal (1997a, 2000). What are sometimes called “self-representational” theories of consciousness (see, e.g., Kriegel (2009)) also hold that if M is conscious then S is aware of M, but they hold that this is because M represents itself. Although some of what follows applies to such theories (see Sect. 3), this paper focuses on HO theories.

5 For appeals to the definition see, e.g., Tye (1997, p. 290), Rosenthal (2000, p. 275), Kriegel (2006, p. 58), Braddon-Mitchell and Jackson (2007, p. 129), Weisberg (2011b, pp. 417–418).

6 Some (Lycan 1996; Hacker 2002; Snowdon 2010) are suspicious of “‘what it is like’-talk”. Although I think none gives us good reason to reject this way of talking, I will not argue for that here (see (Farrell ms.) for a defence of this talk). Instead I will assume, along with most philosophers of mind, that talking in this way is respectable.

7 For example, Block (1997, 2011), Kriegel (2006) and Weisberg (2011b), connect phenomenal consciousness to what-it-is-like-ness. Rosenthal (2011) rejects the connection. Yet other notions of a state’s being conscious are of those states we are conscious *with* (Dretske 1993), and of monitoring and access consciousness (Block 1997).

8 The modest/ambitious distinction is Block’s (2011). Ambitious theorists include Rosenthal (2000), Gennaro (2004) and Weisberg (2011a). Lycan (1996) counts as a modest theorist, but perhaps only in the uninteresting sense that he denies that there is any clear notion of what-it-is-like consciousness that requires explanation.

9 There is an occurrence of what-it-is-like-ness associated with mental state M (e.g., a migraine state) at some time when, at that time, what it is like to be in M (e.g., to have a migraine) contributes to what it is like for the subject overall.

10 Those who have explicitly adopted this stance—in particular, Rosenthal, and Weisberg—are proponents of higher-order *thought* theories of consciousness. But in principle, anyone who favours a HO theory of consciousness requires a response to the misrepresentation argument, and may well be tempted by the awareness argument. And, as noted above, the awareness argument is also endorsed by proponents of self-representational theories of consciousness. Moreover, appeals to the notions of what and something are ubiquitous in philosophical discussions of consciousness. Examining these notions is, then, relevant to the general question of what it is for a mental state to be conscious.

11 An alternative method would be to argue for a particular way of understanding something and what, and see whether it has/lacks the relevant entailments. But, although clarifying exactly how we should understand something and what is an important task, it is also a complicated and controversial matter and one that I have taken up elsewhere (Farrell ms.). (For other attempts, see Hacker (2002), Lormand (2004), Hellie (2004, 2007), Snowdon (2010), Janzen (2011) and Stoljar (2016).) Further, as we’ll see, we don’t need to provide a full account of something or what in order to conclude that ambitious HO theories fail.

12 This is acknowledged by HO theorists, e.g., Rosenthal (1986, 2011) and Weisberg (2011b). Early proponents of arguments from misrepresentation include Byrne (1997) and Neander (1998), see references in Block (2011) for more recent versions of such arguments.

13 Weisberg (2011b) calls these *radical* misrepresentation cases. Non-radical misrepresentation occurs when the subject is in M, but the HO state represents M as being other than it is. We are only concerned with cases in which S is not in M, and so I’ll henceforth drop the ‘radical’.

14 This argument is inspired by that given in Block (2011). It is not clear to me exactly how Block’s argument is intended to go, but it is clear from his paper and the discussion that follows it (Rosenthal 2011; Weisberg 2011b) that the locus of disagreement concerns how we understand something and what. The present statement makes this plain. It also makes plain that the argument doesn’t require (what is false) that a state’s being TP-conscious means the subject is in that state.

15 Thanks to an anonymous referee for emphasising this response. Both Rosenthal (2011) and Weisberg (2011a) offer something like it in reply to Block (2011).

16 An analogous argument replaces what with something and concludes with the negation of (M5). Its first premise is as suspect as (N1) is. Further, it doesn’t show which premise of the valid argument for (M5) must be rejected.

17 It is true that HO theorists accept (M1) because of the way they understand TP-consciousness. But the misrepresentation argument is not sensitive to their reasons for accepting (M1). All that matters insofar as the argument goes is that they do accept (M1).

18 Both Weisberg (2011b, p. 411) and Rosenthal (2011, p. 434) propose a further response to the argument: it assumes that what-it-is-like-ness is a monadic property of M. But the statement of the misrepresentation argument involves no claims about the adicity of what-it-is-like-ness: the argument doesn’t require that ‘M is conscious’ (in (M1) and (M2)) has the logical form *aF*.

19 Cf. the third way of missing the point of the knowledge argument in Lewis (2002).

20 This point doesn’t require that the knowledge argument succeeds, but only that we understand the situation described in Jackson’s thought experiment.

21 To be clear: the difference between the two readings depends on whether there being something it is like for S to be in M entails an occurrence *of what-it-is-like-ness associated with M*. It does not depend on whether there being something it is like for S to be in M entails *an occurrence of M* (i.e., that S is in M).

22 Those who favour the occurrent reading can, of course, describe Mary’s situation. They say that there *would be* something it is like for Mary to see red, and that Mary does not know what it *would be* like for her to see red. But that they must use different words than are standardly used to describe Mary’s situation just emphasises that this reading is non-standard.

23 We might think that proponents of the misrepresentation argument must also adopt the occurrent reading of something as it appears in that argument. But things are more complicated. We don’t yet have the tools to see why this is so, but see footnote 25 below.

24 Note that this doesn’t require that we take the what-it-is-like-ness to be a *property*, let alone a *monadic* property, let alone a monadic property *of M*.

25 We can now see how proponents of the misrepresentation argument can assert the argument whilst adopting a non-occurrent reading of something. They can do this by pointing out that we can legitimately add a third conjunct to (M1). Not only are misrepresentation cases situations in which M is conscious, and S is not in M, they are also (as ambitious HO theorists will acknowledge) cases in which there is an occurrence of what-it-is-like-ness associated with M. If proponents of the argument adopt a tight reading of what, it follows that S is in M, and so the contradiction can still be produced. Can HO theorists avoid the conclusion of this variant of the misrepresentation argument? Just as in the case of the original misrepresentation argument, this comes down to how they understand something and what. What I say in the main text about this applies just as well to the variant version of the argument. Thus I’ll only consider the original version henceforth.

26 Both Rosenthal (2000, p. 275) and Weisberg (2011a, p. 439) endorse this line of reasoning. Non-HO theorists who give the argument include Levine (2007, p. 514), Janzen (2011) and Kriegel (2012, p. 444).

27 As well as those just cited as giving the awareness argument, partisans of (A3) include Church (1995) and Lormand (2004), (and see Janzen (2008, p. 113) for a longer list). Although it is generally agreed that what I (following Rosenthal (2005)) am calling ‘awareness’ is not factive, philosophers may disagree about exactly how (A3) is to be understood. Some may hold, for example, that what S is aware of is not merely *M*, but instead *S’s being in M*. These differences in detail can be ignored in what follows.

28 See, e.g., Thomasson (2008) and Stoljar (2016).

29 See, e.g., Dretske (1995) and Tye (1995).

30 I thank an anonymous referee for suggesting I consider both this and the following explanation of why HO theorists think we should accept (A2).

31 As before, noting that Jackson’s use of something is standard doesn’t require accepting that the knowledge argument is sound

32 Some authors seem to suggest that there is an important distinction between, for example, what it is like to see a tomato, on one hand, and what it is like *for some subject* to see a tomato (see, e.g., Rosenthal (1997b)). But this is not obviously so (see Lormand (2004), Hellie (2007) and Stoljar (2016), and (Farrell ms.)). Either way, it is with claims of the latter—‘for the subject’-involving—kind that we are concerned with.

33 See Hellie’s (2007) discussion of Lormand (2004) for more on this point. See Stoljar (2016) and (Farrell ms.) for discussions of how we should understand this ‘for’, neither of which supports the self-intimating reading.

34 These are Weisberg’s labels (2011a). As we’ll see, they are misleading, but I’ll use them for ease of presentation. Weisberg attributes the zealous reading to Block, although Block denies that this is his view (2011b, p. 420).

35 Rosenthal (2000, p. 275), Janzen (2011, p. 284) and Weisberg (2011b, p. 427).

36 This is sometimes accompanied by the claim that ‘something it is like’ and ‘what it is like’ (when used to talk about consciousness) have a technical meaning which Nagel introduced. This further claim is false (see Farrell 2016), but the provenance argument does not depend on it.

37 Indeed, as we’ll see in Sect. 4.1, Nagel adopts a *non*-self-intimating reading.

38 For a similar statement, see Weisberg (2011b, p. 414).

39 For this to be true, it doesn’t matter whether Nagel is *correct* to think that HO theories cannot account for consciousness. All that matters is that Nagel clearly understands something in a way such that it doesn’t straightforwardly follow *from the very notion* of there being something it is like for S to be in M, that HO theories account for consciousness in this sense. This is compatible with it being true that HO theories *can* account for what-it-is-like consciousness. So we are not begging the question against HO theories here.

40 For both ND(HO) and nd(standard) to both be true, something (on the HO reading) would have to be true when, and only when, something (on the standard reading) was true. But this is not so: the latter can be true in situations in which there is no occurrence of what-it-is-like-ness associated with M; the former cannot. Of course, both ND(HO) and ND(standard) can be true if we take ‘conscious’ to mean different things in these two readings of the definition. But this doesn’t help the HO theorist (see Sect. 4.2 below).

41 See Weisberg (2011b). A natural way of doing this is by holding—as suggested in footnote 40—that ‘conscious’ is to be understood differently as it appears in the HO reading of ND and the standard reading of ND.

42 Thanks to an anonymous reviewer for suggesting this way of understanding this response.
